# The Inhomogeneous Waves in a Rotating Piezoelectric Body

**DOI:** 10.1155/2013/463891

**Published:** 2013-11-05

**Authors:** Xiaoguang Yuan, Si Chen

**Affiliations:** ^1^School of Civil Engineering, Henan Polytechnic University, Jiaozuo 454000, China; ^2^Faculty of Metallurgical and Energy Engineering, Kunming Science and Technology University, Kunming 650093, China

## Abstract

This paper presents the analysis and numerical results of rotation, propagation angle, and attenuation angle upon the waves propagating in the piezoelectric body. Via considering the centripetal and Coriolis accelerations in the piezoelectric equations with respect to a rotating frame of reference, wave velocities and attenuations are derived and plotted graphically. It is demonstrated that rotation speed vector can affect wave velocities and make the piezoelectric body behaves as if it was damping. Besides, the effects of propagation angle and attenuation angle are presented. Critical point is found when rotation speed is equal to wave frequency, around which wave characteristics change drastically.

## 1. Introduction

The gyroscopes of rotating motion sensors have important applications in automobiles, video cameras, smart weapon systems, machine control, robotics, and navigation. Recently, using piezoelectric materials to make vibratory gyroscopes has been of increasing interest. Piezoelectric gyroscopes can make use of rotation-induced frequency shifts in surface acoustic wave (SAW) or bulk acoustic wave (BAW) piezoelectric resonators to measure angular rates. The basic behaviors of a piezoelectric gyroscope are governed by the equations of a rotating piezoelectric body, which consist of the equations of linear piezoelectricity with rotation-related Coriolis and centrifugal accelerations. 

As far as we know, the research of rotation-affected vibration or wave was started by Huston [1] who investigated the effect of “rigid-body” rotation on wave propagation velocities in elastic media. Further, rotation effect was studied in the “in-plane” vibration of rotating circular disks [2]. It was found that the inclusion of Coriolis and centripetal accelerations leads to the result that the medium behaves dispersive and anisotropic [3]. In another contribution [4], the reflection and refraction of plane waves were considered. The effect of rotation on surface acoustic waves was derived theoretically in a perturbation treatment of the Coriolis force for an isotropic medium [5]. The problem of wave propagation in a rotating random infinite magnetothermoviscoelastic medium was studied and a coupled dispersion relation for longitudinal and transverse waves was deduced to determine the effect of viscoelasticity, relaxation times, and rotation on the phase velocity of the coupled waves [6]. In the work of Wauer [7], the propagation of waves in a conducting piezoelectric solid was studied for the case when the entire medium rotates with a uniform angular velocity. Destrade and Saccomandi [8] raised and addressed two questions related to elastic motions and found some finite amplitude transverse waves in rotating incompressible elastic solids with general shear response. Auriault [9] revealed that free wave propagation in non-Galilean rotating media gives rise to two dispersive waves which are coupled dilatational-shear waves. The propagation of surface (Rayleigh) waves over a rotating orthorhombic crystal was studied [10], in which the secular equation for the surface wave speed was found explicitly. In the work of Ting [11], the Stroh formalism for surface waves in an anisotropic elastic half-space was extended to the case when the half-space rotates about an axis with a constant rotation rate. Auriault [12] investigated wave propagation in elastic porous media which are saturated by incompressible viscous Newtonian fluids when the porous media are in rotation with respect to a Galilean frame. Yang [13] presented a review of analyses on the vibrations of rotating piezoelectric structures for applications in piezoelectric angular rate sensors. Propagation of plane waves in a micropolar porous elastic solid rotating with a uniform angular velocity was investigated [14]. The paper [15] dealt with the propagation of body waves in a rotating, generalized thermoelastic solid by using Cardano's and perturbation methods. A two-dimensional problem in electromagnetic micropolar generalized thermoelastic medium subjected to mechanical force or thermal source was investigated [16]. Biryukov et al. [17] investigated the gyroscopic effect in arbitrary crystals by taking into account the medium rotation. Recently, the paper [18] considered the propagation of body waves in a homogenous isotropic, rotating, generalized thermoelastic solid with voids. Wegert et al. [19] analysed theoretical upper bounds for the size of the gyroscopic effect on the frequency of guided acoustic waves in (piezo)elastic media, which are valid in the regime of small rotation rates as compared to the frequency of the guided acoustic wave. The contribution [20] was aimed at the effects of rotation on the propagation of harmonic plane waves under two-temperature thermoelasticity theory. Kothari and Mukhopadhyay [21] analyzed the effects of rotation on the propagation of harmonic plane waves in an unbounded thermoelastic media rotating with a uniform angular velocity. The investigation [22] was performed with the effect of rotation on an infinite circular cylinder subjected to certain boundary conditions. 

As stated above, it is seen that many achievements have been done about the rotation effects on waves. This paper prefers to investigate the rotation on the inhomogeneous waves in piezoelectric body which have been researched by Yuan and his colleagues [23–26]. To our knowledge, no systematic empirical research exists addressing the question of inhomogeneous wave propagation in a rotating piezoelectric body. Our work here is to present the analysis and result for this problem in the framework of inhomogeneous wave theory. 

The paper is organized in the following manner. In the next section, the basic equations for motion in a rotating piezoelectric solid and their wave dispersion equations to harmonic waves are given. Next, using the inhomogeneous wave theory, we recast the dispersion equations in a general complex form which separable real solutions to define the phase velocity and attenuation are admitted. Thus, we can discuss the wave phase velocities, attenuations with three independent parameters: propagation angle, attenuation angle, and rotation speed. Finally, in Sections 3 and 4, numerical results are presented and conclusions are inferred, respectively.

## 2. Basic Governing Equations

**Figure 1:**
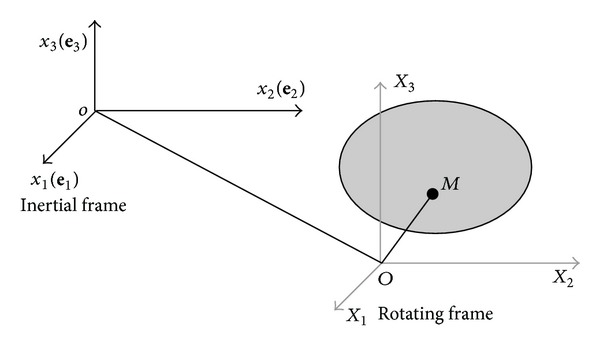
The rotating piezoelectric body.

We consider a linear homogeneous piezoelectric body shown in Figure 1, and *M* is the material point rotating with the speed vector **Ω**( = *Ω*_1_ **e**_1_ + *Ω*_2_ **e**_2_ + *Ω*_3_ **e**_3_). It should be mentioned that throughout this paper, all equations are expressed in the inertial frame *ox*_1_ *x*_2_ *x*_39_, in which there are base vector **e**_1_, **e**_2_, and **e**_3_ along three axes, respectively.

Thus, the momentum balance in a piezoelectric body can be written as

(1) $$\begin{array}{c} \rho \left[\frac{\partial^{2}u}{\partial t^{2}}+\Omega \times \left(\Omega \times u\right)+2\Omega \times \frac{\partial u}{\partial t}\right]=\nabla \cdot \sigma , \end{array}$$

and equivalently, in component form:

(2) $$\begin{array}{c} \rho \left[\frac{\partial^{2}u_{j}}{\partial t^{2}}+\varepsilon_{jik}\varepsilon_{kmn}\Omega_{i}\Omega_{m}u_{n}+2\varepsilon_{jik}\Omega_{i}\times \frac{\partial u_{k}}{\partial t}\right]=\sigma_{ij,i}. \end{array}$$

In the above equation, *ρ* is the mass density, *t* is the time variable, **u** is the displacement vector, **σ** is the Cauchy stress tensor, and *ε*_*jik*_ is the permutation tensor. The subscripts range from 1 to 3. On account of rotation, the term **Ω** × (**Ω** × **u**) denotes the centripetal acceleration, and due to the time-varying motion, 2**Ω** × (∂**u**/∂*t*) corresponds to the Coriolis acceleration [3]. 

Further, the electric field can be described by the electrostatic equation

(3) $$\begin{array}{c} D_{i,i}=0, \end{array}$$

where *D*_*i*_ is the electric displacement vector, and with material equations

(4) $$\begin{array}{c} \sigma_{ij}=C_{ijkl}\varepsilon_{kl}-e_{kij}E_{k},\\ D_{i}=\epsilon_{ij}E_{j}+e_{ikl}\varepsilon_{kl}, \end{array}$$

where *σ*_*ij*_ are the strain tensor and *E*_*k*_ the electric field vector while *C*_*ijkl*_, *e*_*kij*_, and *ϵ*_*ij*_ are the elasticity, piezoelectricity, and permittivity tensors of the material. The Einstein summation is implied in the above equations over the repeated subscripts.

The electric field vector can be derived from an electric potential, that is,

(5) $$\begin{array}{c} E_{k}=-\varphi_{,k}, \end{array}$$

where *φ* is the electric potential. The geometric relationship between the strain and the displacement tensors is defined as

(6) $$\begin{array}{c} \varepsilon_{kl}=\frac{1}{2}\left(u_{k,l}+u_{l,k}\right). \end{array}$$

Eliminating *ε*_*kl*_ and *E*_*k*_ from (4), (5), and (6) yields

(7) $$\begin{array}{c} \sigma_{ij}=C_{ijkl}u_{k,l}+e_{kij}\varphi_{,k},\\ D_{i}=-\epsilon_{ij}\varphi_{,j}+e_{ikl}u_{k,l}. \end{array}$$

Then, instituting (7) into (2) and (3), one obtains a set of wave equations for rotating piezoelectric body

(8) ρ[∂2uj∂t2+εjikεkmnΩiΩmun+2εjikΩi∂uk∂t]  −Cijkluk,li−ekijφ,ki=0  −ϵijφ,ji+eikluk,li=0

which contains four equations (three elastic wave equations and one electric wave equation that is associated with the elastic waves by the material relationship equation (4) and three independent unknown variables: *u*_1_, *u*_2_, *u*_3_, and *φ*). 

## 3. The Inhomogeneous Wave Solutions

Generally, the wave equations of (8) can be solved by introducing complex monochromatic plane wave functions, such as,

(9) $$\begin{array}{c} u_{i}=U_{i}e^{I(k_{j}x_{j}-\omega t)},\\ \varphi =\Psi e^{I(k_{j}x_{j}-\omega t)}, \end{array}$$

where *k*_*j*_ is the complex wave vector, *ω* is the wave circular frequency, *I* is the imaginary unit $\left(I=\sqrt{-1}\right)$, *t* is the time variable, and (*U*_*i*_, Ψ) are the complex amplitudes of displacements and electric potential, respectively. Inserting (9) into (8) gives

(10) ρω2[−Uj+εjikεkmnΩiωΩmωUn−2IεjikΩiωUk]  +CijklUkklki+ekijkkkiΨ=0,ϵijkjkiΨ−eiklUkklki=0.

A nontrivial solution of these four linear homogeneous equations for *U*_1_, *U*_2_, *U*_3_, and Ψ exists only if the determinant of the coefficients vanishes, which yields the governing dispersion relation

(11) $$\begin{array}{c} \det G=0, \end{array}$$

in which the elements *g*_*ij*_  (*i*, *j* = 1, 2, 3, 4) of the matrix **G** are

(12) $$\begin{array}{c} \left[\begin{array}{cccc} g_{11} & g_{12} & g_{13} & g_{14}\\ g_{21} & g_{22} & g_{23} & g_{24}\\ g_{31} & g_{32} & g_{33} & g_{34}\\ g_{41} & g_{42} & g_{43} & g_{44} \end{array}\right]\left\{\begin{array}{c} U_{1}\\ U_{2}\\ U_{3}\\ \Psi \end{array}\right\}=\left\{\begin{array}{c} 0\\ 0\\ 0\\ 0 \end{array}\right\}, \end{array}$$

where

(13) g1s=ρω2[−δ1s+ε1ikεkmsΩiωΩmω−2Iε1isΩiω]+Ci1slklki, s=1,2,3,g14=eki1kkkiΨ,g2s=ρω2[−δ2s+ε2ikεkmsΩiωΩmω−2Iε2isΩiω]+Ci2slklki, s=1,2,3,g24=eki2kkkiΨ,g3s=ρω2[−δ3s+ε3ikεkmsΩiωΩmω−2Iε3isΩiω]+Ci3slklki, s=1,2,3,g34=eki3kkkiΨ,g41=−ei1lklki,  g42=−ei2lklki,g43=−ei3lklki,  g44=ϵijkjki.

Further, with the help of inhomogeneous wave theory [23, 25], assume that the complex wave vector can be decomposed in terms of wave propagation direction as

(14) $$\begin{array}{c} k_{j}=P_{j}+iA_{j}=Pn_{j}+iAm_{j}, \end{array}$$

where *P*_*j*_ is the propagation vector with its magnitude of $P=\sqrt{P_{j}P_{j}}$, *A*_*j*_ is the attenuation vector with its magnitude of $A=\sqrt{A_{j}A_{j}}$, and (*n*_*j*_, *m*_*j*_) are the unit vectors along the propagation direction (normal to the equiphase plane) and the perpendicular to the plane of constant amplitude (normal to the equiamplitude plane), respectively. Generally, *n*_*j*_ ≠ *m*_*j*_ represents an inhomogeneous wave problem while *n*_*j*_ = *m*_*j*_ represents a special case of a homogeneous wave problem.

**Figure 2:**
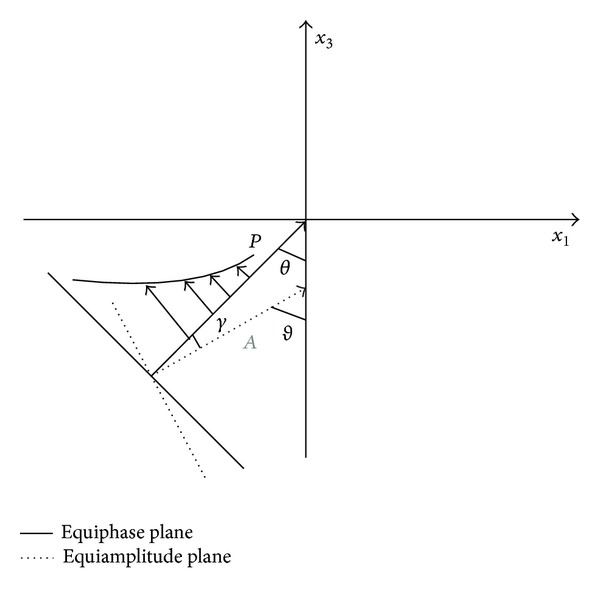
Illustration of equiphase and equiamplitude planes and exponential variation of the amplitude along the phase propagation direction.

Further, the unit vectors (*n*_*j*_, *m*_*j*_) can be further expressed in terms of the angle *θ* between *n*_*j*_ and *x*_3_, the angle *γ* between *n*_*j*_ and *m*_*j*_ as shown in Figure 2. Via (14), we obtain

(15) $$\begin{array}{c} \left\{n_{1},n_{2}\right\}={\left\{\sin\theta ,\cos\theta \right\}}^{T},\\ \left\{m_{1},m_{2}\right\}={\left\{\sin\left(\theta +\gamma \right),\cos\left(\theta +\gamma \right)\right\}}^{T},\\ n_{j}m_{j}=\cos\gamma . \end{array}$$

Correspondingly, the wave vector *k*_*i*_ can be expressed in terms of one complex number, the propagation angle *θ*, and the attenuation angle *γ*, such that,

(16) $$\begin{array}{c} k_{1}=P\sin\theta +iA\sin\left(\theta +\gamma \right),\\ k_{2}=P\cos\theta +iA\cos\left(\theta +\gamma \right). \end{array}$$

Inserting (16) into the dispersion equation (11) and then decomposing it into the real and imaginary parts leads to solvable equations in terms of *P* and *A* for the given attenuation angle *γ*, propagation angle *θ*, and rotation speed **Ω**

(17) DR(P,A)=0,DI(P,A)=0, A∈0∪R+,

where *D*_*R*_ and *D*_*I*_ are the operators on *P* and*A*, which are nonlinear and coupled algebraic equations in terms of (*P*, *A*). According to the definitions of *P* and *A* in (14), the right solution of *P* and *A* should be real-valued. Therefore, the domain of attenuation angle *γ* is determined by the condition that *P* and *A* are nonnegative real numbers (here only the positive direction of wave propagation is considered). Thus, the wave propagates with the phase velocity *c*_*p*_ and the nonnegative attenuation *A*, which agrees with the Sommerfeld radiation condition, that is, vanishing at infinity. Generally, there are three roots of (*P*, *A*) that are related to three elastic wave modes: one quasilongitudinal (QL) and two quasitransverse (QT 1, 2) waves for the given *θ*, *γ*, and **Ω**. It is noted that, due to the static electric field assumption, there is no independent wave mode in the electric field, whereas the electric wave still can propagate with the elastic wave modes via the constitutive relationship (4). After *P* is solved, the phase velocity can be defined as

(18) $$\begin{array}{c} c_{p}=\frac{\omega }{P}, \end{array}$$

and *A* is the corresponding wave attenuation.

## 4. Results and Discussions

In order to discuss the problem in greater detail and to find out the effects of the rotation speed **Ω** of the body, propagation angle *θ*, attenuation angle *γ* on the phase speed *c*_*p*_, and attenuation coefficient *A* of the inhomogeneous wave, we have computed them by taking the following piezoelectric material parameters in Table 1. All the physical constants are rewritten with the help of Voigt notation, whose rule is that the subscripts of a tensor are transformed by the rule {11 → 1,22 → 2,33 → 3,23 → 4,31 → 5,12 → 6}. 

For convenience, a parameter *K*_*i*_ can be defined as

(19) $$\begin{array}{c} K_{i}=\frac{\omega }{\left|\Omega \right|},\\ \Omega =\Omega_{1}e_{1}+\Omega_{2}e_{2}+\Omega_{3}e_{3}, \end{array}$$

which is used to discuss the effects of rotation speed vector on the phase velocity and attenuation. Also the direction of rotation speed vector along *x*_3_, *x*_1_ will be considered and compared in the following. The wave frequency *ω* here is set to be 2*π* × 10^6^ 1/second.

**Figure 3:**
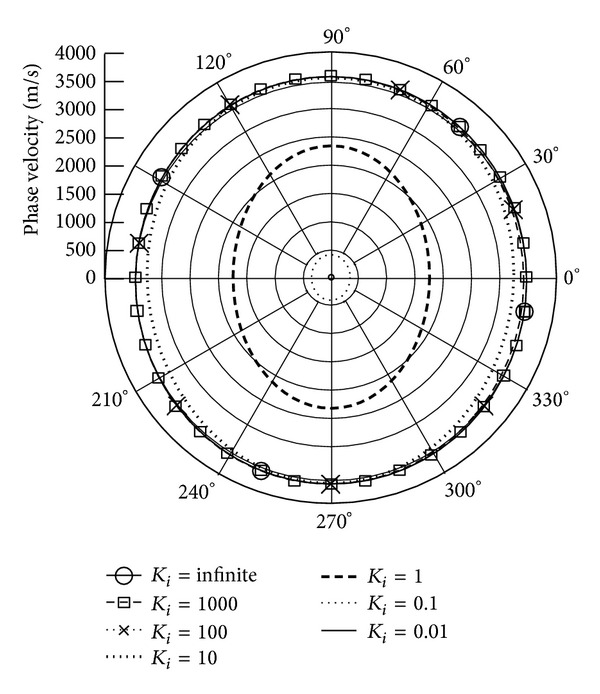
Phase velocity of quasitransverse wave (QT1) versus propagation angle *θ* ranging from 0° to 360° with *γ* = 0 and varied *K*_*i*_, when **Ω**  =  *Ω*_3_ **e**_3_.

**Figure 4:**
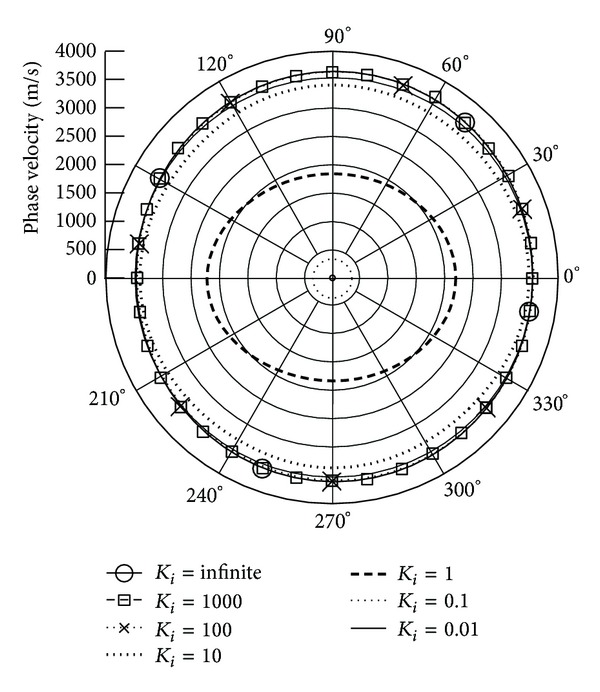
Phase velocity of quasitransverse wave (QT1) versus propagation angle *θ* ranging from 0° to 360° with *γ* = 0 and varied *K*_*i*_, when **Ω**  =  *Ω*_1_ **e**_1_.

*(I) The Phase Velocity.* Figures 3 and 4 illustrate the phase velocity of QT1 wave when the piezoelectric body rotation about the *x*_3_ and *x*_1_ axes with varied rotation speeds and *γ* = 0, respectively. The data show that the rising rotation speed leads to declining phase velocities. Because of the anisotropic property of piezoelectric body, the phase velocity performs differently at different propagation angles. It can be seen that there is a sharp drop in phase velocity at *K*_*i*_ = 1; that is, the rotation speed is equal to the wave frequency; at the same time, the rotation direction influences the velocities. When *K*_*i*_ is below 1 or the rotation speed is more than wave frequency, the velocity slope is larger than when *K*_*i*_ is above 1. It is found that the attenuation angle *γ* almost does not influence the phase velocity.

**Figure 5:**
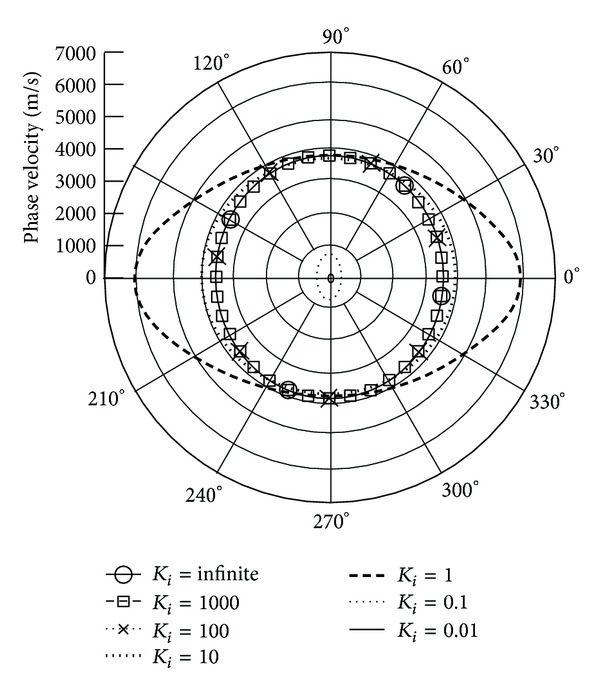
Phase velocity of quasitransverse wave (QT2) versus propagation angle *θ* ranging from 0° to 360° with *γ* = 0 and varied *K*_*i*_, when **Ω**  =  *Ω*_3_ **e**_3_.

**Figure 6:**
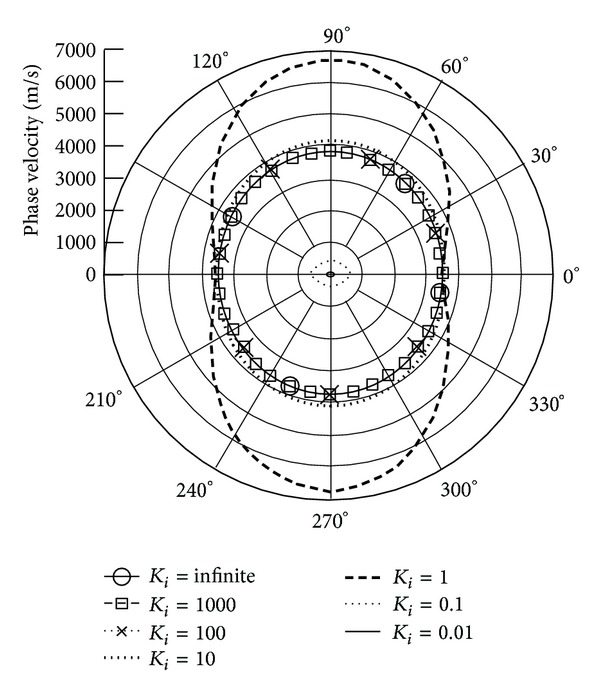
Phase velocity of quasitransverse wave (QT2) versus propagation angle *θ* ranging from 0° to 360° with *γ* = 0 and varied *K*_*i*_, when **Ω**  =  *Ω*_1_ **e**_1_.

The cases of quasitransverse wave QT2 are found in Figures 5 and 6, which is similar with QT1, except that the phase velocity of *K*_*i*_ = 1 is larger than that of other values of *K*_*i*_ at most propagation angles.

**Figure 7:**
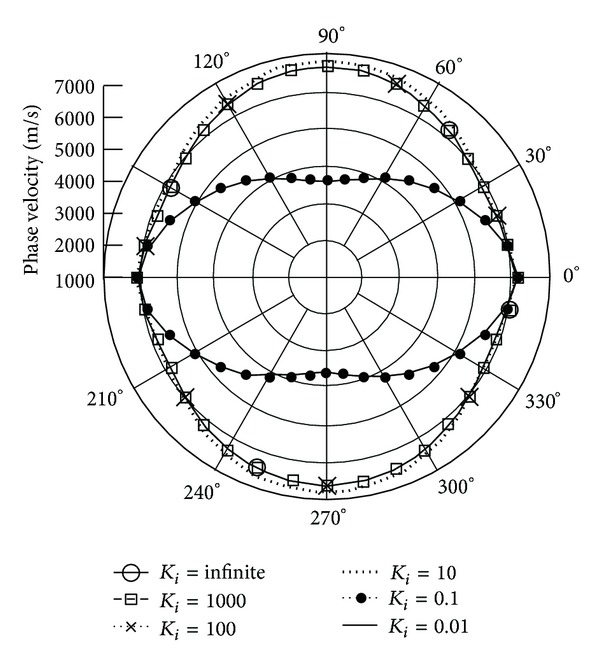
Phase velocity of quasilongitudinal wave (QL) versus propagation angle *θ* ranging from 0° to 360° with *γ* = 0 and varied *K*_*i*_, when **Ω**  =  *Ω*_3_ **e**_3_.

**Figure 8:**
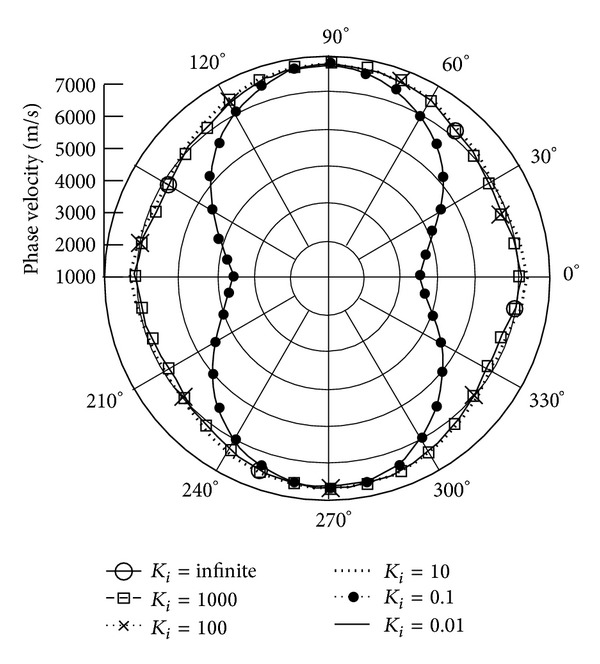
Phase velocity of quasitransverse wave (QL) versus propagation angle *θ* ranging from 0° to 360° with *γ* = 0 and varied *K*_*i*_, when **Ω**  =  *Ω*_1_ **e**_1_.

Figures 7 and 8 illustrate the quasilongitudinal wave (QL) velocities along *x*_3_, *x*_1_ axes. The data suggest that there is no quasilongitudinal QL wave in the case of *K*_*i*_ = 1 (when rotation speed is equal to wave frequency). Rather than QT1, 2, the phase velocities of *K*_*i*_ = 0.1, 0.01 and *K*_*i*_ = infinite, 1000, 100, 10 are very close.

**Figure 9:**
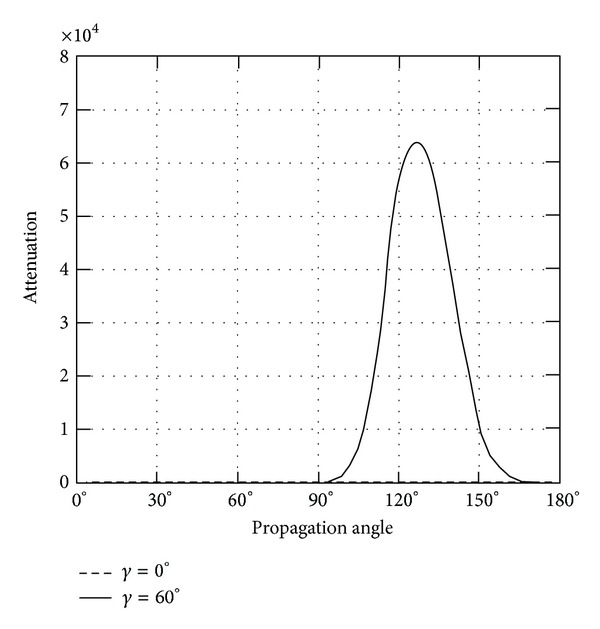
Attenuation of quasitransverse wave (QT1) versus propagation angle angle *θ* ranging from 0° to 180° with *γ* = 0°, 60°, when *Ω*_3_ = 2*π* × 10^8^ or *K*_*i*_ = 0.01.

**Figure 10:**
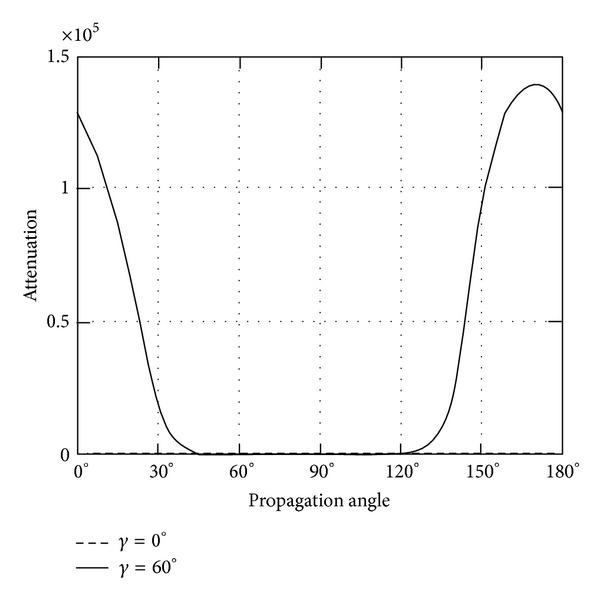
Attenuation of quasitransverse wave (QT1) versus propagation angle *θ* ranging from 0° to 180° with *γ* = 0°, 60°, when *Ω*_1_ = 2*π* × 10^8^ or *K*_*i*_ = 0.01.

**Figure 11:**
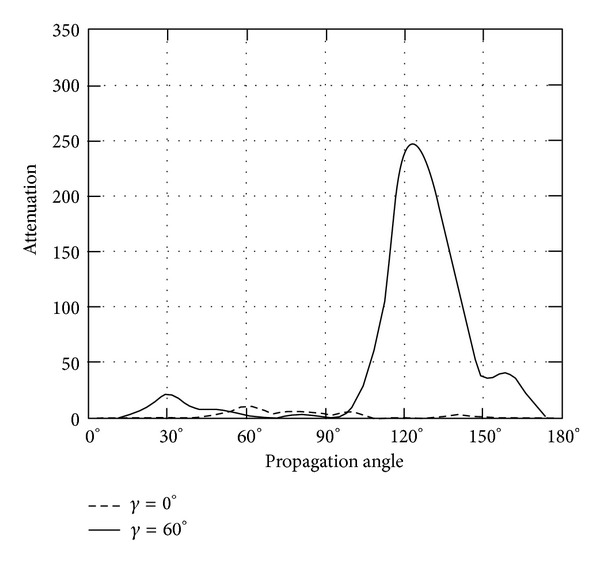
Attenuation of quasitransverse wave (QT2) versus propagation angle *θ* ranging from 0° to 180° with *γ* = 0°, 60°, when *Ω*_3_ = 2*π* × 10^8^ or *K*_*i*_ = 0.01.

**Figure 12:**
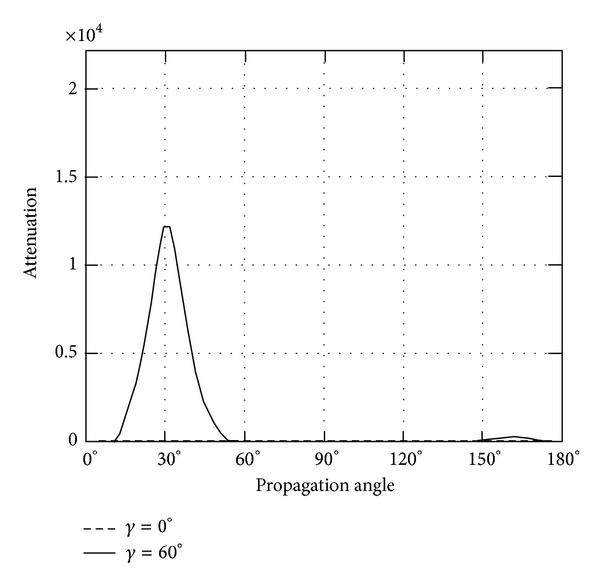
Attenuation of quasitransverse wave (QT2) versus propagation angle *θ* ranging from 0° to 180° with *γ* = 0°, 60°, when *Ω*_1_ = 2*π* × 10^8^ or *K*_*i*_ = 0.01.

**Figure 13:**
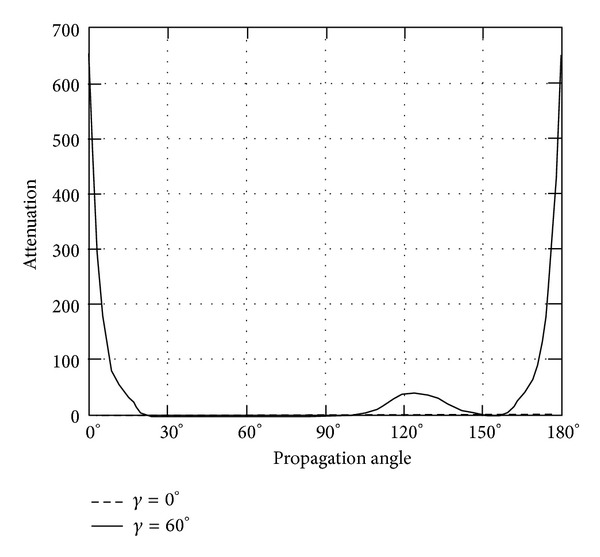
Attenuation of quasilongitidinal wave (QL) versus propagation angle *θ* ranging from 0° to 180° with *γ* = 0°, 60°, when *Ω*_3_ = 2*π* × 10^8^ or *K*_*i*_ = 0.01.

**Figure 14:**
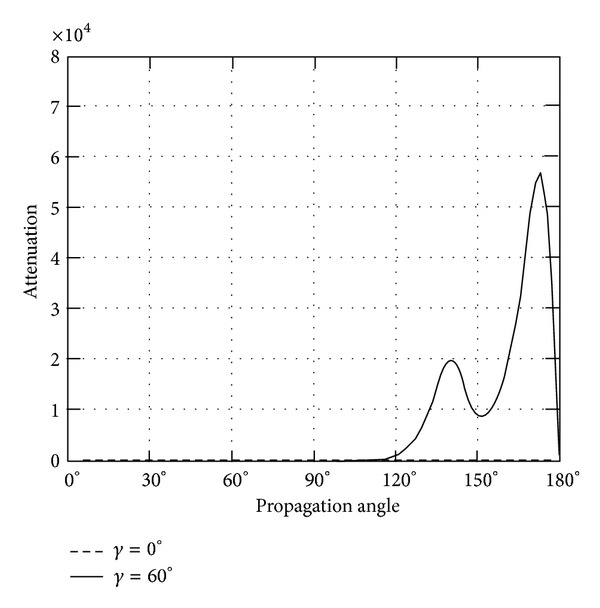
Attenuation of quasilongitidinal wave (QL) versus propagation angle *θ* ranging from 0° to 180° with *γ* = 0°, 60°, when *Ω*_1_ = 2*π* × 10^8^ or *K*_*i*_ = 0.01.

*(II) The Attenuation*. Instead of phase velocity, the attenuation angle *γ* influences the wave attenuation notably, which can be demonstrated in Figures 9–14. These figures imply that only large rotation speed or small *K*_*i*_ can affect wave attenuation, and attenuation angle can amplify such effect significantly. Therefore, the cases of *K*_*i*_ = 0.01 are taken to demonstrate the attenuation angle influences.

Figures 9 and 10 depict the wave attenuations of QT1 wave mode. It is seen that when attenuation angle *γ* = 0 of homogeneous wave, no attenuation is found to exist for any rotation speed; only when the attenuation angle is above zero, that is *γ* = 60, the wave attenuation gains sharply around propagation angle *θ* = 140 for piezoelectric body rotating around *x*_3_ and 0, 180 for rotating around *x*_1_. With the same value of rotation speed, the rotation around *x*_1_ axis shows more impact on the wave attenuation than that around *x*_3_ axis.

Turning back to QT2 wave, it is revealed that there is slight oscillating when attenuation angle *γ* = 0. Likewise, nonzero attenuation angle plays large roles, which are shown in Figures 11 and 12.

Further, the attenuations of quasilongitudinal wave (QL) are shown in Figures 13 and 14. The largest increase in attenuation is found at propagation angle *θ* = 0, 180 for rotating about *x*_3_ axis. The attenuation of *γ* = 60 jumps when propagation angle is above 120, when the body rotates about *x*_1_.

## 5. Conclusions

In the framework of inhomogeneous wave theory, the propagation of waves in rotating piezoelectric solid has been analyzed firstly. Throughout this paper, four independent parameters are used to study the waves of rotating body, which are rotation speed value and its direction, propagation angle, and attenuation angle. The obtained results demonstrate that the rotation speed influences the wave characteristics significantly. It is found that *K*_*i*_ = 1 is the critical point, that is, when rotation speed is equal to the wave frequency, around which the phase velocity varies substantially; at this point, no quasilongitudinal wave exists. Even though no damping parameters are taken into account, still tiny wave attenuation can be induced by the rotation speed. The large rising attenuations are attributable to attenuation angle. If there is no rotation, the attenuations of QT1, QT2, and QL waves are found to be zero. It is also noted that the wave velocity, as well as attenuation, behaves differently for different directions of rotation speed vector.

## Figures and Tables

**Table 1:** Material properties of Ba_2_NaNb_5_O_15_ crystal.

*C*_*ij*_ (GPa)	*C*_11_	*C*_22_	*C*_33_	*C*_44_	*C*_55_	*C*_66_	*C*_12_	*C*_13_	*C*_23_

	239	247	135	65	66	76	104	50	52

*e*_ij_ (C·m^−2^)	*e*_31_	*e*_32_	*e*_33_	*e*_24_	*e*_31_	*e*_31_			

	−4.0	−0.3	4.3	3.4	−0.4	4.3			

*ϵ*_*ij*_	*ϵ*_11_/*ϵ*_0_	*ϵ*_22_/*ϵ*_0_	*ϵ*_33_/*ϵ*_0_						

*ϵ*_0_ = 8.854 × 10^−12^ F/m	222	227	32						

*ρ* (kg/m^3^)									

	5,700								
